# Moisture Sorption Isotherms and Thermodynamic Properties of Biodegradable Polymers for Application in Food Packaging Industry

**DOI:** 10.3390/polym15071634

**Published:** 2023-03-24

**Authors:** Loleny Tavares, Liliana R. Sousa, Sara Magalhães da Silva, Paulo S. Lima, J. M. Oliveira

**Affiliations:** 1 School of Design, Management and Production Technologies Northern Aveiro, University of Aveiro, Estrada do Cercal, 449, 3720-509 Oliveira de Azeméis, Portugal; 2 EMaRT Group – Emerging: Materials, Research, Technology, School of Design Management and Production Technologies Northern Aveiro, University of Aveiro, Estrada do Cercal, 449, 3720-509 Oliveira de Azeméis, Portugal; 3 CICECO Aveiro-Institute of Materials, Campus Universitário de Santiago, University of Aveiro, 3810-193 Aveiro, Portugal; 4 TEMA, Centre for Mechanical Technology and Automation, Mechanical Engineering Department, Campus Universitário de Santiago, University of Aveiro, 3810-193 Aveiro, Portugal

**Keywords:** biopolymers, thermoplastic, starch, food packaging, thermodynamics, biodegradable

## Abstract

This work aims to evaluate the influence of two starch-based materials (B16 and B20) on the moisture sorption isotherms, determined at 30, 40, and 50 °C, where B16 contains 5% (w/w) more starch than B20. Thermodynamic functions (differential enthalpy (∆H_dif_), differential entropy (∆S_dif_), integral enthalpy (Δh_int_), integral entropy (ΔS_int_), free Gibbs energy (∆G), and spreading pressure (φ)) were used to understand the water-binding behaviors and the energy requirements to remove the moisture content from the surface of these materials. The moisture sorption isotherms exhibited type III behavior, and the Guggenheim–Anderson–de Boer (GAB) model was the most suitable to fit the experimental moisture adsorption data. The adsorption isotherms of microparticles were enthalpy-controlled, with isokinetic temperature values of 221.45 and 279.77 K for B16 and B20, respectively, being higher than the harmonic mean temperature (312.94 K). The values of ∆G were positive (45.274 and 44.307 kJmol^−1^ for B16 and B20, respectively), indicating a non-spontaneous process. The spreading pressure values increased with increasing water activity (aw) for all isotherms. Higher values of ∆H_dif_ and ∆S_dif_ obtained from B16 confirmed its higher number of sorption sites available for binding with water molecules when compared to B20, making it less suitable for application in the food packaging industry.

## 1. Introduction

Industrialization and changes in consumer habits have increased the utilization of plastics in different industries, and currently, the global plastic production in 2020 is around 359 million tons, with a revenue of approximately EUR 350 billion for the European plastics sector [1,2]. Plastic production has increased exponentially since the 1950s, and products from plastics have become indispensable in our daily lives due to their accessibility, lightweight, relatively low price, durability, and excellent moldability [3,4]. Despite its usefulness and benefits, less than 10% of the plastic produced is subjected to recycling processes; 78% is disposed of in landfills or leaks into the environment, and only 12% goes to incineration, creating massive environmental pollution [5]. The use of bio-based (obtained from renewable resources) or biodegradable (subjected to microbial degradation under aerobic (composting) or anaerobic conditions) plastics are sustainable alternatives to their petrochemical counterparts to reduce the enormous volume of non-biodegradable plastic waste [6,7]. Based on the key differences between the terms “bio-based” and “biodegradable” polymers [8], bio-based and biodegradable polymers include polylactic acid (PLA), polysaccharides derivatives, poly(amino acid), and polyhydroxyalkanoates (PHA); petrochemical biodegradable polymers include polybutylene succinate (PBS), poly(butyl acrylate) (PBA), poly(butylene succinate-co-butylene adipate) (PBSA), polycaprolactone (PCL), and poly(butylene adipate-co-terephthalate) (PBAT). Poly(butylene adipate-co-terephthalate) (PBAT) is widely used for the manufacture of biodegradable food packing due to its good processability properties (e.g., high elongation and excellent flexibility) [9,10]. However, PBAT is an expensive polymer that costs around twice to three times more than conventional plastics such as polyethylene, which is widely utilized in the packaging industry [11,12]. Native starch is abundant in nature and commonly used in different industries due to its low-cost resources obtained from various agro-industrial wastes; thermoplastic starch (TPS), produced from native starch using different plasticizers (glycerol, sorbitol, and water, among others), has been used to produce biodegradable and bio-based packaging in food industries [13,14]. The cost of TPS is around one-twentieth that of PBAT. The cost of producing PBAT packaging is significantly decreased by blending it with starch while maintaining a high level of biobased carbon [9].

Different studies have demonstrated that native starch films used in the food packaging industry possess certain drawbacks, such as brittleness, poor thermal processability, and hydrophilicity, which strongly impact the mechanical properties and water vapor barrier characteristics of plastic packaging materials [14,15]. To overcome these problems, different methodologies have been studied to improve thermoplastic starch, including acetylation, octenyl succinic anhydride (OSA)-modification, cross-linking, hydroxypropylation, oxidation and blending of TPS with other polymers [16]. Even though several techniques for altering native starch have been developed in recent years to produce plastic packaging materials, its tendency to absorb moisture and its poor mechanical properties continue to be difficult to overcome fully [17]. It is well known that the data from sorption isotherms can be used to investigate structural aspects of food products, such as specific surface area and pore radius, as well as to anticipate the ideal storage conditions and determine the shelf-life stability of a product [18]. In this sense, this work aims to understand the water adsorption isotherm, thermal, and diffusion behavior of two biopolymers containing different wt% of starch, identified as B16 and B20, respectively. For this purpose, the sorption isotherm, thermodynamic sorption behavior, surface area, and pore sizes under different temperatures were studied to evaluate the effects of moisture content, water activity, temperature, and starch composition on the properties of the two starch-based biodegradable polymers.

## 2. Materials and Methods

**Figure 1:**
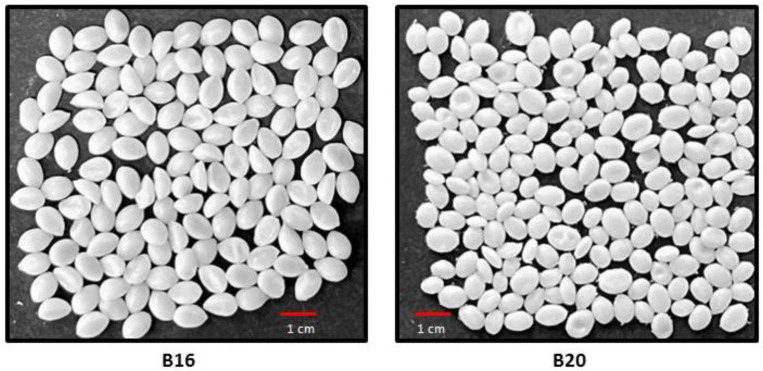
B16 and B20 granules. Scale bar is 1 cm.

The starch-based biodegradable polymer B16 contains 5% (w/w) more starch than B20. The product data sheet for B16 includes a melt flow rate (MFI) of 6.6 g/10 min (130 °C, 10 kg) and a density of 1.29 g/cm^3^. For B20, it includes an MFI of 4.6 g/10 min (130 °C, 10 kg) and a density of 1.28 g/cm^3^. B16 and B20 granules are represented in Figure 1.

### 2.1. Fourier-Transform Infrared Spectroscopy (FTIR)

A Fourier-transform infrared spectroscopy (FTIR) technique was used to obtain the infrared spectrum of absorption of the B16 and B20 samples. FTIR spectra were collected on a Bruker INVENIO S FTIR spectrometer equipped with an attenuated total reflection (ATR) attachment using platinum ATR (A225/Q) with a diamond crystal plate as an accessory. For each sample, the infrared (IR) spectra were recorded in the range of 4000–500 cm^−1^ at a resolution of 4 cm^−1^ and 64 scans.

### 2.2. Water Adsorption Isotherms

The water adsorption isotherms of the starch-based biodegradable polymers (B16 and B20) were determined using the COST 90 Project’s static gravimetric approach, evaluated at 30, 40, and 50 °C [1]. The experiment involved 9 salt-saturated aqueous solutions (barium chloride, copper sulfate, potassium carbonate, magnesium chloride, sodium nitrite, potassium chloride, sodium chloride, and lithium chloride), which were used in order to obtain a relative humidity range between 11 and 98%. A small glass desiccator with a clamp lid was filled with one gram of each sample and each of the aforementioned saturated salt solutions and was stored in hermetically sealed desiccators placed in a hot air oven (Binder, FD 260, Tuttlingen, Germany) until a state of equilibrium was reached (approx. 21 days) [2]. Different mathematical models Equations (1)–(5) (Table 1), were applied to fit the absorption data, and statistica software (version 8.0) was used to determine the models’ parameters.

The degree of fit of the various models was evaluated based on the results of the mean relative percentage deviation modulus (*E*) and coefficient determination (R^2^). The *E* was calculated using Equation (6):

(6) $$E(\%)=\frac{100}{N}\sum_{i=1}^{N}\frac{\left|X_{\mathrm{ei}}{-X}_{\mathrm{pi}}\right|}{X_{\mathrm{ei}}}$$

where *X_pi_* and *X_ei_* are the predicted and measured equilibrium sorption moisture content and *N* is the number of available data points.

### 2.3. Data Analysis of Thermodynamic Properties

#### 2.3.1. Net Isosteric Heat of Sorption

The Clausius–Clapeyron equations (Equations (7) and (8)) were used to calculate the differential enthalpy Δ*h_dif_* (J·mol^−1^), which is the amount of energy associated with the sorption process that is present above the temperature of heat of vaporization of water (Δ*H_vap,_* J·mol^−1^) [3]:

(7) $${\left[\frac{\partial {\;\ln(a}_{w})}{\partial \;\frac{1}{T}}\right]}_{X_{e}}=-\frac{q_{\mathrm{st}}}{R}$$

and,

(8) $${\Delta h}_{\mathrm{dif}}{=q}_{\mathrm{st}}{+\Delta H}_{\mathrm{vap}}$$

where *T* is the absolute temperature (K), *q_st_* (J·mol^−1^) is the net isosteric heat of sorption, *X_e_* the equilibrium moisture content (g water·g^−1^ dry basis), and *R* is the universal gas constant (8.314 J·mol^−1^·K^−1^). The Δ*H_vap_* (J·mol^−1^) at 40 °C (43,345 J·mol^−1^) was used for subsequent analysis and corresponded to the mean temperature of 30–50 °C [4]. Afterward, ln(a_w_) versus 1/T was used to plot the experimental sorption data, the *q_st_* values were obtained from the slope (−*q_st_*/*R*), and the Δ*h_dif_* was determined by applying Equation (8).

The intercept of the line of ln *a_w_* versus 1/*T*, which is represented by Equation (9), was used to calculate the differential entropy (Δ*S_dif_*) at a specific moisture content [5]:

(9) $${\left(\ln a_{w}\right)}_{X_{e}}=\frac{{-\Delta H}_{\mathrm{dif}}}{\mathrm{RT}}+\frac{\Delta S_{\mathrm{dif}}}{R}$$

#### 2.3.2. Enthalpy–Entropy Compensation Theory

The compensation theory proposed a linear relationship between differential enthalpy (Δ*h_dif_*) and differential entropy (Δ*S_dif_*), as shown in Equation (10):

(10) $$\Delta h_{dif}=T_{\beta }\cdot \Delta S_{dif}+\Delta G$$

where Δ*G* is the Gibbs free energy (J·mol^−1^) and *T_β_* denotes the isokinetic temperature (K). The enthalpy–entropy compensation theory was verified and validated according to Krug, Hunter, and Grieger [6] through a comparison of the *T_β_* value with the mean harmonic temperature *T_hm_* (K) value, represented in Equation (11):

(11) $$T_{hm}=\frac{n}{\sum_{1}^{n}\left(\frac{1}{T}\right)}$$

where *n* represents the overall count of sorption isotherms employed.

#### 2.3.3. Spreading Pressure

The spreading pressure (*φ* in J·m^2^) shows the surface excess free energy and indicates how sorbed molecules raise the surface tension of the bare sorption sites [7]. The values of *φ* were determined according to Equation (12) [8]:

(12) $$\varphi =\frac{K_{B}\times T}{A_{m}}\times \int_{0}^{aw}\frac{X_{e}}{X_{m}\times a_{w}}\times da_{w}$$

where *K_B_* is the constant of Boltzmann (1.380 × 10^−23^ J·K^−1^), *X_e_* is the equilibrium moisture content, *A_m_* is the surface area of a water molecule equal to 1.06 × 10^−19^ m^2^, and *X_m_* is the monolayer moisture content. At a value of *a_w_* = 0, the spreading pressure’s value is unknown, and as a result, the lowest limit was determined to be *a_w_* = 0.05 [8].

The spreading pressure was calculated by integrating and rearrangement of the GAB model, as represented in Equation (13):

(13) $$\varphi =\frac{K_{B}\times T}{A_{m}}\times \ln{\left[\frac{1+C\times k\times a_{w}-k\times a_{w}}{1-k\times a_{w}}\right]}_{0.05}^{a_{w}}$$

where *C* and *k* are the GAB constants at each temperature. The constants *d*, *e*, and *f* were determined from the values of φ obtained from Equation (13), obtaining Equation (14):

(14) $$\varphi =\frac{d\cdot e\cdot f\cdot a_{w}}{\left(1-f\cdot a_{w}\right)\times (1-f\times a_{w}+e\cdot f\cdot a_{w})}$$

#### 2.3.4. Integral Enthalpy and Entropy

The integral enthalpy (Δ*h_int_*, J·mol^−1^) was determined similar to the methodology used in Equation (7); however, maintaining the value of φ constant instead of the moisture content (Equation (15)) [7]:

(15) $${\left[\frac{\partial \;\ln\left(a_{w}\right)}{\partial \;\frac{1}{T}}\right]}_{\varphi }=-\frac{\Delta h_{int}}{R}$$

The values of Δ*h_int_* were found by re-plotting the experimental sorption data in the form of ln(*a_w_*) versus 1/*T*, for a specific value of φ. The integral entropy (Δ*S_int_*, J·mol^−1^ K) was then calculated according to Equation (16) [9]:

(16) $$\Delta S_{int}=-\frac{\Delta h_{int}}{T}-R\cdot \ln\left(a_{w}{}^{*}\right)$$

where a_w*_ is the geometric mean water activity at a constant value of φ determined at 30, 40, and 50 °C.

#### 2.3.5. Specific Surface Area and Pore Size Analysis

The surface areas and pore sizes were analyzed using the results of isotherms data. The specific surface area *S*_0_ (m^2^·g^−1^) was calculated using Equation (17) [10], with values of the monolayer moisture content *X_m_* (g water/g dry basis) determined by the GAB model,

(17) $$S_{0}=X_{m}\cdot \frac{1}{M_{H_{2}O}}\times N_{0}\cdot A_{H_{2}O}=3.5\times 10^{3}\times X_{m}$$

where $M_{H_{2}O}$ is the molecular weight of water (18 g·mol^−1^), $A_{H_{2}O}$ is the area of a water molecule (10.6 × 10^−20^ m^2^), and *N_0_* is the number of Avogadro (6 × 10^23^ molecules·mol^−1^).

The *R_p_* pore radius (nm) was calculated as the result of adding the *r_c_* critical radius (nm) and *t* multilayer thickness (nm). The values of *r_c_*, *t*, and *R_p_* values were evaluated by applying the following Kelvin and Halsey’s equations, namely Equations (16), (18)–(20), respectively [11].

(18) $$r_{c}=\frac{2\cdot \gamma \cdot V_{M}}{R\cdot T\cdot \ln\left(\frac{1}{a_{w}}\right)}$$

(19) $$t=0.354\cdot {\left(\frac{-5}{\ln a_{w}}\right)}^{\frac{1}{3}}$$

(20) $$R_{p}=t+r_{p}$$

where *γ* is the surface tension of the water (J·m^−2^), *R* is the universal gas constant (8.134 J·mol^−1^·K^−1^), *V_M_* is the molar volume of water as sorbate (1.8·10^−5^ mol^−1^ m^3^), and *T* is the temperature of service (K).

The *a_w_* values for each temperature and moisture content were determined by using the GAB model (Equation (21)):

(21) $$a_{w}=\frac{2+\left(\frac{X_{m}}{X_{e}}-1\right)\cdot C-{\left\{{\left[2+\left(\frac{X_{m}}{X_{e}}-1\right)\cdot C\right]}^{2}-4\cdot \left(1-C\right)\right\}}^{0.5}}{2\cdot K\cdot \left(1-C\right)}$$

where *X_m_* represents the monolayer moisture content on a dry basis, i.e., the amount of water necessary for one water molecule to completely saturate each primary adsorption site; *k* is a correction parameter that reflects the characteristics of multilayer molecules in relation to the bulk liquid; and *C* is the Guggenheim constant, representing the energy difference between the water molecules bonded to the primary sorption sites [12].

### 2.4. Statistical Analysis

The SAS software (version 9.3) was used to analyze the statistical significance. Analysis of variance (ANOVA) and the Tukey test (*p* < 0.05) were used to compare the means of the data. Statistica software (version 8.0) was used to carry out the regression analysis.

## 3. Results and Discussion

### 3.1. FTIR Spectra

**Figure 2:**
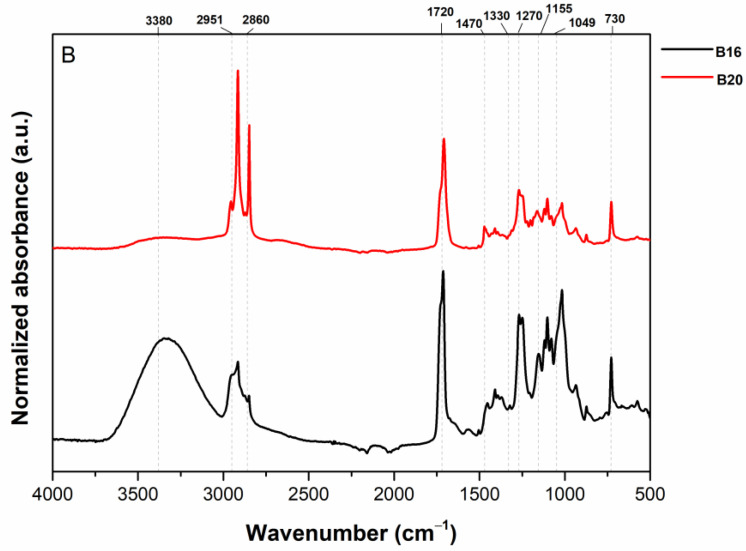
FTIR spectra of B16 and B20 samples.

The attenuated total reflectance–Fourier-transform infrared spectroscopy (ATR-FTIR) technique was used to assess the molecular structure, chemical bonds, and changes that can occur during the moisture sorption isotherm of two starch-based materials (B16 and B20) (Figure 2). The starch molecular structure consists of amylopectin and amylose, which have α-1,4- and α-1,6-linked glucose units, respectively [13]. The following peaks were identified in both samples: broad absorption bands between 3000 and 3600 cm^−1^ were attributed to the complex vibrational stresses associated with the hydroxyl groups’ chemical bonds (free, inter-, and intra-molecular) [14]. Two weak peaks at 2951 cm^−1^ and 2860 cm^−1^ indicated the C–H stretching of the glucose ring, and the peak at 1720 cm^−1^ indicated the presence of C = O bonds. The peak at 1420 cm^−1^ was associated with CH_2_ vibrations, 1330 cm^−1^ (CH bending), 1270 cm^−1^ (C–O–C stretching), 1150 cm^−1^ (C−O−C bonds), ~1049 cm^−1^ (C−O stretching), and 730 cm^−1^ (bending modes of C–H bonds in aromatic rings). B16 and B20 samples showed absorption peaks in the same wavenumber regions, which revealed that the two samples possessed similar functional groups. However, B16 showed higher peak intensities between 3000 and 3600 cm^−1^ than B20, indicating a molecular structure with more hydroxyl groups available to interact with polar water molecules when exposed to high relative humidity environments. B20 exhibited a higher intensity of peaks at 2951 cm^−1^ and 2860 cm^−1^ than B16, indicating more available C–H groups that were not much affected by moisture content. Starch is highly hygroscopic; the degree of moisture absorption is affected by the hydrogen bonds formed by the hydroxyl groups of the aminoglycoside units along the chain [15]. In this sense, the results of FTIR spectra can be used to adjust the amount of starch content in the polymer formulation in order to absorb a known quantity of moisture content, which is essential for progressing research into starch-based materials for food packaging applications.

### 3.2. Water Sorption Isotherm

**Figure 3:**
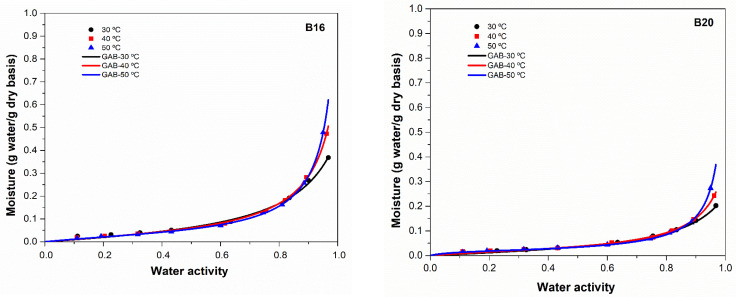
Experimental and estimated (Gab model) moisture adsorption data as a function of water activity and temperatures of 30, 40, and 50 °C.

The results of water sorption isotherms can provide important information regarding the interactions between the hydrophilic active functional groups of polymers and the polar water molecules, which enables one to predict the product’s stability and shelf life during the storage process [16,17]. There are five sorption isotherm models that are frequently employed to correlate the experimental moisture adsorption data, namely GAB, Halsey, Smith, Oswin, and Peleg models. Table 1 presents the parameters of each model for both starch-based biodegradable polymers at 30, 40, and 50 °C. The GAB and Peleg models are the best ones to describe the experimental adsorption data, considering the higher values of coefficient determination (*R*^2^) and the lowest values of average relative deviation (*E*). However, the Peleg model fails to describe the sorption isotherm at 50 °C for both samples, considering that the values of *E* exceeded 10%, while the GAB model presents values of *E* lower than 10% for all isotherms [18]. In addition, the GAB model has been recommended by the European Project Group on COST 90 as the fundamental model to determine the moisture content present in the materials [19]. In this sense, the GAB model was chosen to evaluate the water sorption isotherms for the two starch-based biodegradable samples (Figure 3).

Analyzing the GAB estimated parameters, the monolayer moisture content (*X_m_*) values decreased with increased temperature for both samples. These results demonstrated the excitation of water molecules at higher temperatures, which reduced the contact between the polar water molecules and water molecules/polymer, lowering the water sorption on the surface of the polymer [20]. These results also reflected a decrease in the number of active sites caused by the increased temperature, which led to a breakaway from the water-binding sites and structural, physicochemical changes [21]. The values of *X_m_* were higher for B16 than B20 in all the three isotherms studied (30, 40, and 50 °C), indicating a higher number of polar sites on the surface of B16 to interact with water molecules. The higher amount of starch present in B16 also influences its structure and its capacity to interact with other molecules. As the monolayer moisture content describes the quantity of water strongly adsorbed to particular surface sites [22], these values can be used to define the best storage conditions of both B16 and B20. The obtained values of *K* were less than 1, suggesting that the water molecules on the monolayer surface were more tightly bound than those present in the surface multilayer [23]. In addition, the liquid sorbate in the multilayers was found to be in a pure state since the k values were near to 1 [24].

The GAB model’s constant C is related to the net enthalpies of monolayer sorption [25]. This parameter controls the water molecules’ binding energy to the main binding sites on the product’s surface and relates to the potential energy difference between the monolayer and the higher layers. The greater its value, the more tightly the water molecules in the monolayer are bound to the binding sites on the surface of the sorbent [26]. With rising temperatures, the values of constant *C* increased, and the adsorption on polymer surfaces became less intensely localized. The B20 exhibited higher values of *C* than B16, indicating that the polar water molecules were more tightly bonded to the surface of the polymer. In addition, the values of *C* increased with increasing temperature from 30 to 50 °C. This result may be related to the material superficial hardness, which happens when the rate of superficial evaporation exceeds the rate of moisture diffusion inside the surface of the product, changing its physical structure by obstructing its pores [27,28]. Viollaz and Rovedo [29] reported sorption data for starch at four temperatures, obtaining the following GAB parameters: *X_m_* values of 0.10221, 0.09828, 0.08573, and 0.08738; k values of 0.78696, 0.78848, 0.79277, and 0.71900; and C values of 28.4147, 18.9935, 18.0858, and 11.3984 for 2.7 °C, 20.2 °C, 40.2 °C, and 67.2 °C, respectively. Moghaddam et al. [30] produced blends of polylactic acid plasticized with acetyl tributyl citrate and thermoplastic wheat starch (TPS), reporting an increasing X_m_ value with an increased TPS ratio due to its hydrophilic nature. These authors also reported that the increase in TPS content favors the sample’s surface ability to absorb more water molecules. The equilibrium moisture adsorption data for the B16 and B20 are represented in Figure 3. The B16 and B20 samples exhibited typical Type III sigmoid adsorption isotherms, where the characteristic shape of both isotherms showed low values of moisture content at low a_w_ and increased sharply at high a_w_ [31]. These results suggest that both polymers have micropores and macropores on their surface, and the water vapor adsorption occurs mainly due to capillary condensation. It is known that the product surface has highly active polar sites with high interaction energies during the early stages of sorption (low moisture content), and these sites are available to be covered with polar water molecules to create a monomolecular surface layer [26]. Therefore, as the water molecules bind chemically to these highly active sites, sorption takes place at less active sites (with high moisture content).

### 3.3. Differential Enthalpy and Entropy

**Figure 4:**
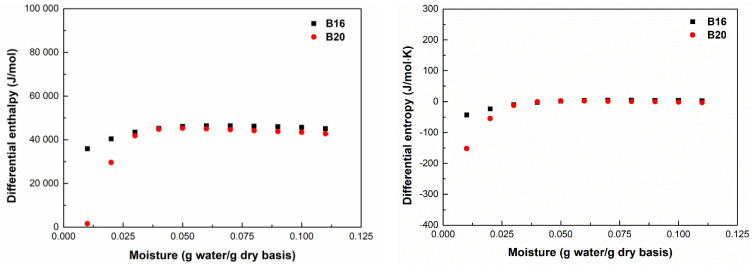
Differential enthalpy and entropy values of adsorption as a function of equilibrium moisture contents for B16 and B20.

The differential enthalpy (∆*h_dif_*) and differential entropy (Δ*S_dif_*) as a function of the equilibrium moisture content for B16 and B20 are shown in Figure 4. The ∆*h_dif_* increased as moisture content increased. Until moisture contents of 6% and 5% are reached for B16 and B20, respectively, there is an increase in ∆*h_dif_*, then a decline, until the curve becomes nearly asymptotic. These results suggest the existence of residual water molecules inside the pore networks of polymers, and thereby, less energy to remove them. When this amount of residual water was removed, more energy was necessary to remove other water molecules that were more strongly attracted to the active hydrophilic polar groups on the surface. At a moisture content higher than 5%, the values of ∆*h_dif_* for both samples approached the heat of water vaporization, indicating the point at which water molecules exist in free form in the product [37]. The heat of sorption for B16 was higher than that of B20, demonstrating that the higher starch concentration affected the amount of water that the polymer’s surface could absorb. The sorption ∆*S_dif_* of both samples increased with increasing *X_e_* values. Changes in ΔS*_dif_* can be related to the variation in the number of available sorption sites [20]. For lower *X_e_*, the water molecules were found within the high-energy binding sites on the material surface, allowing for minimal rotational freedom and degree of randomness, thus leading to lower entropies. When the values of *X_e_* increased, the high-energy sites started to saturate, which allowed an increase in the mobility of water molecules adsorbed and, consequently, the degree of randomness and entropy increased. Moreover, a high moisture content has a plasticizing impact that increases the freedom of the water molecules in the surface structure and raises entropy due to the mobility of macromolecules and their free volume [20,38]. The quantity of available sorption sites on the surface at a given energy level is inversely correlated with a material’s differential entropy [39]. In this sense, the higher values of ∆*S* values obtained from B16 indicate a structure with a hydrophilic surface with a high number of available sorption sites to interact with polar water molecules.

### 3.4. Enthalpy–Entropy Compensation

**Figure 5:**
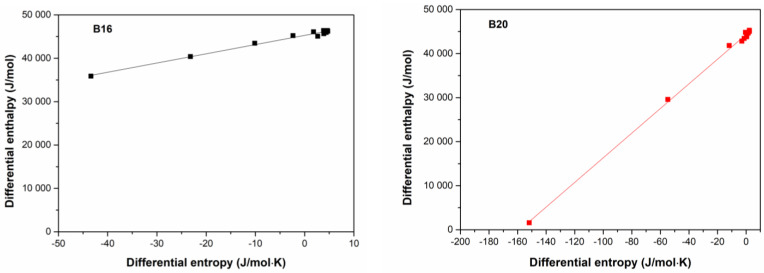
Enthalpy–entropy compensation plots for B16 and B20.

The enthalpy–entropy compensation theory was evaluated as a result of the linear relationship between Δ*h_dif_* as a function of Δ*S_dif_* (Figure 5). The following two linear equations were obtained: Δ*h_dif_* = 221.45 × Δ*S_dif_* + 45,274 (R^2^ = 0.9864) for B16 and Δ*h_dif_* = 279.77 × Δ*S_dif_* + 44,307 (R^2^ = 0.9984) for B20. The linear relationship presented coefficients of determination (*R^2^*) higher than 0.98 for the adsorption processes in both materials. The values of isokinetic temperature (*T_β_*) and Gibbs free energy (Δ*G*) parameters obtained in the linear regression for B16 and B20 curves were 221.45 K and 45.274 kJ mol^−1^, and 279.77 K and 44.307 kJ mol^−1^, respectively. The harmonic mean temperature (*T_hm_*) obtained was 312.94 K. The value of *T_hm_* ≠ *T_β_* confirmed the compensatory theory. According to Leffler [40], if *T_β_* > *T_hm_* or *T_β_* < *T_hm_*, the process is enthalpy-driven or the is entropy controlled, respectively. According to the obtained results, the enthalpy phenomena controls the sorption for both samples. The positive values of ΔG suggested a non-spontaneous process of water adsorption.

### 3.5. Spreading Pressure

**Figure 6:**
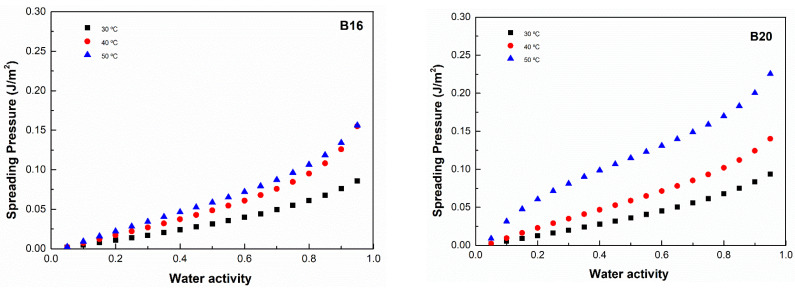
Spreading pressure adsorption isotherms for B16 and B20 at 30, 40, and 50 °C.

The spreading pressures of B16 and B20 at different *a_w_* and each working temperature are shown in Figure 6. The results show that the spreading pressure decreased with increasing values of water activity and temperatures. At each operating temperature, the B20 had larger spreading pressure readings than the B16, indicating a surface with a high excess of free energy. This result was mainly affected by the higher values of parameter *C* obtained in the Gab model, resulting in higher calculated values of *a_w_*. Similar behavior of spreading pressures was reported by Tao et al. [22].

### 3.6. Integral Enthalpy and Entropy

**Figure 7:**
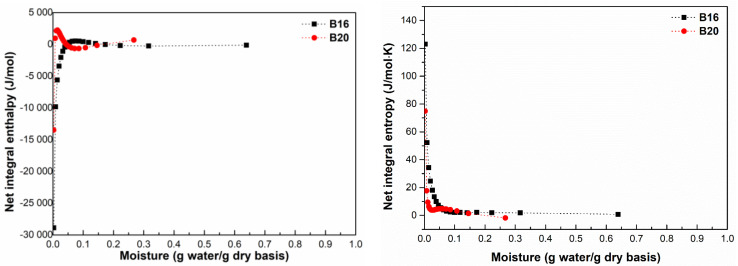
Net integral enthalpy and entropy of adsorption as a function of equilibrium moisture content for B16 and B20.

The net integral enthalpy is a sign of the strength potential of water molecules to interact with the surface of the material [22,41]. The values of integral enthalpy (Δ*h_int_*) and integral entropy (Δ*S_int_*) as a function of the moisture content for B16 and B20 are shown in Figure 7. The Δ*h_int_* values for both samples increased significantly when the moisture content increased, achieving a maximum value of 531.959 J·mol^−1^ (*X_e_* = 0.074 g water·g^−1^ db) and 2232.595 J·mol^−1^ (*X*_e_ = 0.021 g water·g^−1^ db) for B16 and B20, respectively. Then, the Δ*h_int_* values gradually decreased with increasing of *X_e_*; the smallest Δ*h_int_* values at lower *X_e_* suggested that water molecules were occupying the most available sites on the external surface of the material and that water–solid interactions were more relevant than the water–molecule interactions [26,42]. For B20, the interaction between its polar hydrophilic active sites and water molecules showed a higher value of maximum Δ*h_int_* and stronger binding strength than B16 at lower *X_e_*.

The Δ*S_int_* values of both samples dropped as moisture content increased until a plateau of 0.1 g water·g^−1^ db was attained. The decrease in the Δ*S_int_* represents a limitation in the water molecules’ motion due to the strongest binding sites between the surface and water molecules [4,26,43]. The plateau showed that the moisture content was getting close to being saturated, and the values of Δ*S_int_* tended toward the entropy of free energy of liquid water (0 J/mol K) [9].

At a moisture content value of <0.13 g water·g^−1^ db, the absolute Δ*S_int_* values for B20 were lower than that obtained for B16, proving its low ability to interact with water molecules during the adsorption procedure [22].

According to the result, both polymers must be stored in an environment with an *X_e_* range below 0.10 g water·g^−1^, where high absolute Δ*S_int_* values are found. At this range, since the water molecules are well organized on the material’s surface, they are less molecules suitable to participate in unwanted reactions [22].

### 3.7. Surface Area and Porosity

The surface areas and pore sizes of B16 and B20 were determined using the experimental adsorption isotherm data. The values of surface area (*S*_0_) for B16 and B20 samples were evaluated according to Equation (17). The *S*_0_ values determined at 30, 40, and 50 °C were 224.0, 164.5, and 133 m^2^g^−1^ for B16 and 115.5, 87.5, and 66.5 m^2^g^−1^ for B20. The total surface area suitable to interact with water molecules decreased with increasing temperature. The *S*_0_ values were higher for B16 than B20 due to its surface having a hydrophilic nature that contained a high number of active polar sites available to interact with polar water molecules.

The pore radius (*Rp*) for B16 ranged from 0.92 to 7.18 (temperature of 30 °C), 0.87 to 6.62 nm (temperature of 40 °C), and 0.83 to 6.57 nm (temperature of 50 °C), and for B20 ranged from 1.08 to 35.27 nm (temperature of 30 °C), 0.96 to 17.62 nm (temperature of 40 °C), and 0.76 to 12.93 nm (temperature of 50 °C), with moisture contents varying from 0.01 to 0.2 g water·g^−1^ db (Table 2). The surface of both polymers contains micropores and mesopores, taking into account the IUPAC classification, where pores are classified into the following groups: micropores < 2.0 nm, mesopores range between 2.0 and 50 nm, and macropores > 50 nm [31]. The *Rp* values increased as the values of temperature and moisture content increased. Since the temperature impacts the rate of entry and exit of water molecules, the pore diameters typically increase as temperatures and moisture contents increase [26,44,45]. The results of *S_0_* and *Rp* are very important to predict the best storage conditions since the available sites and pore sizes of the material determines the overall sorption surface area, which affects how quickly and thoroughly water molecules hydrate its surface [46,47].

## 4. Conclusions

The temperature, amount of starch, moisture content, specific surface area, and mean pore diameter influenced the results of moisture sorption isotherms. FTIR results indicated that the peak intensity associated with the free, inter-, and intra-molecular bonds of OH hydroxyl groups increased with increased starch content, indicating a molecular structure with more hydroxyl groups available to interact with polar water molecules when exposed to high relative humidity environments. The equilibrium moisture content decreased as water activity decreased at a constant temperature. These characteristics are important to establish the proper handling and storage conditions to extend shelf life and to select the most suitable properties of the packaging materials. The GAB model provides the best fitting for the adsorption isotherms, showing higher values of coefficient of determination (R^2^) and lower values of the mean relative percentage deviation modulus (*E*) in comparison with the other studied models. The results of thermodynamic properties revealed that the adsorption isotherms of both polymers was enthalpy-controlled, with isokinetic temperature values higher than the harmonic mean temperature. Polymers contain micropores and mesopores on their surface, and their adsorption rate depends on their open pore structure, temperature, and moisture content in the monolayer. 

Both B16 and B20 samples must be stored in an environment with a moisture content below the obtained values of monolayer moisture content (ranging from 0.019 to 0.064) to avoid physical and chemical degradation, which can occur at a relative humidity of 1.9 and 6.4% d.b. The nitrogen adsorption isotherm technique could be applied in future studies to determine the surface area and pore size of samples in order to compare the effect of the water molecule size (0.2 nm) and nitrogen molecule size (0.43 nm) on penetrating the surface structure of the samples. The results of the moisture isotherm and thermodynamic properties provide important knowledge about the best parameter conditions to be used during the drying and storage process of starch-based biodegradable biopolymers, for application in the food packaging industry.

## Figures and Tables

**Table 1:** Estimated values of coefficients evaluated using different models for the isotherms of B16 and B20 at 30, 40, and 50 °C.

			Temperature (°C)					
			30		40		50	
Model	Equation	Constant	B16	B20	B16	B20	B16	B20
**GAB**	$X_{e}=\frac{C\cdot K\cdot X_{m}\cdot a_{w}}{(1-K\cdot a_{w})\times (1{-K\times a}_{w}+C\cdot K\cdot a_{w})}$ (Equation (1)) [32]	*X_m_*	0.064	0.033	0.047	0.025	0.038	0.019
		*C*	1.602	1.986	2.359	3.839	3.358	15.506
		*k*	0.873	0.875	0.940	0.934	0.972	0.981
		*R* ^2^	0.997	0.997	0.999	0.999	0.999	0.999
		*E* (%)	9.153	9.037	9.249	9.998	5.624	3.604
**Halsey**	$X_{e}={\left(\frac{-A}{{\ln\;a}_{w}}\right)}^{\frac{1}{B}}$ (Equation (2)) [33]	*A*	0.005	0.002	0.013	0.004	0.021	0.009
		*B*	1.937	1.994	1.525	1.652	1.263	1.274
		*R* ^2^	0.971	0.978	0.991	0.993	0.998	0.999
		*E* (%)	35.878	29.697	30.192	17.553	18.696	3.104
**Smith**	$X_{e}=A+(B\cdot {\log(1-a}_{w}))$ (Equation (3)) [34]	*A*	−0.003	0.002	−0.028	−0.007	−0.037	−0.015
		*B*	−0.251	−0.134	−0.325	−0.162	−0.344	−0.186
		*R* ^2^	0.994	0.997	0.983	0.986	0.966	0.952
		*E*	126.29	116.51	161.75	132.96	179.363	155.013
**Oswin**	$X_{e}=A\cdot {\left[\frac{a_{w}}{{1-a}_{w}}\right]}^{B}$ (Equation (4)) [35]	*A*	0.081	0.046	0.071	0.043	0.058	0.033
		*B*	0.463	0.448	0.593	0.544	0.716	0.712
		*R* ^2^	0.981	0.987	0.995	0.997	0.999	0.998
		*E (%)*	23.306	18.285	13.618	8.887	6.011	14.304
**Peleg**	$X_{e}=k1\cdot \;a_{w}{}^{n1}+k2\;\cdot \;a_{w}{}^{n2}$ (Equation (5)) [36]	*k*1	0.074	0.057	0.507	0.250	0.647	0.085
		*k*2	0.356	0.179	0.123	0.070	0.165	0.424
		*n*1	0.542	0.703	9.322	9.470	13.591	1.048
		*n*2	5.695	6.490	1.022	0.840	1.422	15.514
		*R* ^2^	0.999	0.999	0.998	0.999	0.999	0.997
		*E (%)*	3.14	5.44	7.04	7.47	13.71	11.38

**Table 2:** Pore radius (nm) of B16 and B20 at temperatures of 30, 40, 50 °C and moisture content ranging from 0.01 to 0.2 g·water/g dry basis (db).

Moisture Content (g Water/g Dry Basis)	B16			B20		
	Temperature (°C)			Temperature (°C)		
	30	40	50	30	40	50
0.01	0.92	0.87	0.83	1.08	0.96	0.76
0.02	1.16	1.11	1.06	1.48	1.34	1.15
0.03	1.39	1.34	1.31	1.90	1.79	1.72
0.04	1.61	1.58	1.57	2.37	2.28	2.31
0.05	1.84	1.83	1.84	2.88	2.81	2.90
0.06	2.09	2.08	2.12	3.44	3.37	3.50
0.07	2.34	2.35	2.41	4.06	3.97	4.10
0.08	2.60	2.62	2.70	4.76	4.60	4.71
0.09	2.88	2.90	3.00	5.54	5.28	5.33
0.1	3.16	3.19	3.30	6.42	6.01	5.95
0.2	7.18	6.62	6.57	35.27	17.62	12.92

## Data Availability

The data presented in this study are available on request from the corresponding authors.
